# The Influence of Dietary Habits and Physical Activity on Quality of Life of Peripheral Arterial Disease in Patients Hospitalized at the Department of Vascular Surgery and Transplantation, Medical University in Białystok

**DOI:** 10.3390/nu18050784

**Published:** 2026-02-27

**Authors:** Łukasz Stypułkowski, Michał Chlabicz, Mateusz Jadeszko, Maciej Chlabicz, Sylwia Joanna Barańska, Sławomir Ławicki, Jerzy Bertrandt, Jerzy Głowiński

**Affiliations:** 1 Faculty of Medicine with the Division of Dentistry and Division of Medical Education in English, Medical University of Białystok, ul. Jana Kilińskiego 1, 15-089 Białystok, Poland; 2 Department of Vascular Surgery and Transplantation, Medical University of Białystok, ul. Jana Kilińskiego 1, 15-089 Białystok, Poland; michal.chlabicz@umb.edu.pl (M.C.); mjadeszko96@gmail.com (M.J.); mchlabicz@protonmail.com (M.C.); baranskasj@gmail.com (S.J.B.); jglow@wp.pl (J.G.); 3 Department of Population Medicine and Lifestyle Diseases Prevention, Medical University of Bialystok, ul. Jana Kilińskiego 1, 15-089 Białystok, Poland; slawicki@umb.edu.pl; 4 Faculty of Economic Sciences, John Paul II University in Biala Podlaska, Sidorska 95/97, 21-500 Biala Podlaska, Poland; jwbertrandt@gmail.com

**Keywords:** peripheral arterial disease, quality of diet, physical activity, health-related quality of life, educational level, vitamin supplements, lifestyle interventions

## Abstract

**Introduction**: Peripheral arterial disease is a chronic condition and a major public health concern. Risk factors of PAD include poor dietary habits, low physical activity levels, smoking tobacco and coexisting diseases like hypertension, diabetes or hyperlipidemia. The goal of the study was to evaluate the influence of dietary habits, physical activity and selected quality-of-life factors on PAD among patients hospitalized at the Department of Vascular Surgery and Transplantation, Medical University of Białystok. **Methods:** The study was conducted on 127 patients diagnosed with PAD. Standardized questionnaires were used: IPAQ (short version) to assess physical activity, FFQ-6 to evaluate of the quality of diet and SF-36 to evaluate health-related quality of life. **Results:** A positive correlation between the educational level and diet quality was found—higher education associated with a greater proportion of high-quality products in diet (*p* = 0.001). A negative correlation was found between age and physical activity level (*p* < 0.001). Physical activity level was associated with differences in the SF-36 physical component summary, with statistically significant differences observed between the categories of activity (*p* = 0.047). The positive influence of vitamin supplements on the SF-36 mental component summary was not found; patients taking vitamin supplements had worse MCS results. On top of that, higher physical activity was associated with lower MCS, and longer time spent sitting with higher MCS. **Conclusions:** The findings suggest that the lifestyle interventions for PAD patients should consider their educational level and the age-related decrease in physical activity. Physical activity remains significantly associated with the physical component of quality of life. Concurrently, the PCS observations suggest that only intensifying physical activity or supplementation is not necessarily associated with the improvement of mental wellbeing. Further analysis that accounts for clinical status and possible confounding factors are warranted.

## 1. Introduction

Peripheral arterial disease, also referred to as PAD, is a chronic disease of atherosclerotic origin, leading to the gradual narrowing or full closure of the lumens of the arterial vessels of the lower limbs. The disease constitutes a substantial public health issue, being both a sign of an advanced disease of the cardiovascular system, and a risk factor for thromboembolic complications and amputations [1,2].

The most significant modifiable risk factors of PAD include poor dietary habits, low physical activity levels, smoking tobacco and coexisting diseases like hypertension, diabetes or hyperlipidemia. In recent years, increasing importance has been attached to the assessment of patients’ lifestyle in the context of both primary and secondary prevention of cardiovascular diseases [1,3,4,5].

Peripheral artery disease (PAD) substantially impairs patients’ ability to perform activities of daily living, thereby reducing quality of life [6]. Among symptomatic patients, intermittent claudication is the most commonly reported complaint. Rest pain, however, typically occurs in more advanced stages of the disease [2,7]. In addition, patients often report sleep disturbances, which are associated with worsening lower-limb pain at night [2]. According to current guidelines, a key component of non-pharmacological management is regular, targeted exercise therapy, which improves walking performance and quality of life [2,8]. Dietary modification toward a Mediterranean dietary pattern and comprehensive cardiovascular risk reduction are also recommended and are associated with a more favorable prognosis and a lower burden of risk factors in patients with PAD [8,9].

The effectiveness of PAD management depends not only on pharmacotherapy and surgical or endovascular interventions, but also on adherence to lifestyle recommendations, including physical activity and dietary habits. In everyday clinical practice, difficulties are frequently observed in implementing dietary recommendations and increasing physical activity levels among patients with PAD.

In light of the evidence from the literature, the objectives of this study were to characterize the study population in terms of sociodemographic characteristics and lifestyle factors, including dietary habits and level of physical activity. Additionally, health-related quality of life and lipid profile parameters were assessed. The simultaneous assessment of these domains in a population of hospitalized patients with PAD may provide clinically relevant data and complement the existing literature.

## 2. Materials and Methods

### 2.1. Study Characteristics

The study had an observational, cross-sectional design. It was conducted among 127 patients with a diagnosis of lower-extremity peripheral artery disease (PAD), hospitalized in the Department of Vascular Surgery and Transplantation, Medical University of Białystok, between 8 January 2024 and 28 January 2025. The overall study duration (from the start of recruitment to completion of data collection) was 16 months. For each patient, questionnaire data (IPAQ, SF-36, FFQ) and laboratory results (HDL, LDL, triglycerides, total cholesterol) were collected during hospitalization. The study cohort comprised 95 men and 32 women, with a median age of 70 years.

Inclusion criteria were as follows: age ≥ 18 years; clinically and imaging-confirmed PAD; and provision of written informed consent. Exclusion criteria included the following: lack of informed consent; inability to reliably complete the questionnaires (e.g., significant cognitive impairment/altered mental status or communication barriers precluding history taking); and severe clinical condition preventing completion of the questionnaire assessment during hospitalization. These exclusions were applied to minimize information bias and to ensure comparability of data for statistical analyses.

Of the 127 patients, 93 had complete biochemical data (total cholesterol, LDL, HDL, triglycerides), whereas 34 were evaluated based on questionnaire data only.

### 2.2. Research Tools

#### 2.2.1. Physical Activity—IPAQ Questionnaire

Evaluation of the physical activity was based on the International Physical Activity Questionnaire (IPAQ—short version), which allows for estimation of the energy expenditure expressed in MET—minutes/week [10,11]. Participants reported the number of days and the average duration of vigorous, moderate, and walking activities during the past 7 days. Based on these data, MET-minutes per week (MET-min/week) were calculated separately for each activity category using the formula MET × minutes/day × days/week, applying the standard IPAQ coefficients: 3.3 METs for walking, 4.0 METs for moderate-intensity activity, and 8.0 METs for vigorous-intensity activity. The results for the three domains (walking, moderate, and vigorous activity) were then treated as components of total physical activity. Total physical activity was calculated by summing the MET-min/week values for walking, moderate-intensity, and vigorous-intensity activity, yielding a final outcome expressed as total MET-min/week. Data were processed in accordance with the IPAQ guidelines for data checking and standardization to minimize the impact of extreme values and improve comparability across participants. Based on the obtained data, patients were classified into one of three categories:

- Low physical activity: <600 MET-min/week;
- Moderate physical activity: 600–3000 MET-min/week;
- High physical activity: >3000 MET-min/week [11].

#### 2.2.2. Dietary Habits—FFQ-6 Questionnaire

The diet was evaluated based on the polish version of Food Frequency Questionnaire FFQ-6, containing 62 groups of nutritional products [12,13]. The analysis was conducted based on a set of 20 products deemed especially important from the perspective of cardiovascular disease prevention. Products have been grouped into three categories:

- High-quality products (vegetables, fruits, wholegrain bread, thin meat, dairy with low fat content, plant oils, nuts, fatty fish);
- Moderate-quality products (eggs, milk, white bread, cream, margarine, yellow cheese);
- Low-quality products (ex. sweets, salty snacks, confectionery, fizzy drinks, alcohol, butter, lard, sauces, sweet fruit preserves).

The FFQ-6 questionnaire with an 8-point food-frequency response scale was used. To enable quantitative analysis of dietary data, FFQ-6 responses were converted into an arbitrary intake-frequency score (point weights) and used to construct a diet quality index. Each frequency category was assigned a numerical value, increasing with higher reported consumption. The weights were fixed for all participants and preserved the ordinal structure of the categories (higher category = higher weight). The assigned values were operational and intended solely for within-sample comparisons; they should not be interpreted as the actual number of servings per day. The conversion of intake-frequency categories into point weights is presented in Table 1.

For each patient, the weights were summed within product-quality groups (good-/mediocre-/poor-quality), and the percentage contribution of high-quality products relative to all included products was then calculated. Based on this percentage, patients were assigned to one of three diet quality categories (Table 2). Operational interpretative cut-offs were applied to categorize dietary patterns within the study sample. A value of >60% was considered to indicate a clear predominance of high-quality products in the overall dietary profile (“good diet”), 40–60% indicated a mixed profile (“mediocre diet”), and <40% indicated a low contribution of high-quality products (“poor diet”). These thresholds were used for within-sample comparisons and relative interpretation and should not be regarded as population norms.

#### 2.2.3. Quality of Life Evaluation—SF-36 Questionnaire

For subjective health evaluation, the SF-36 (Short Form Health Survey) questionnaire was used, consisting of 36 items. The survey comprises eight subscales describing different areas of functioning: physical functioning (PF), role limitations due to physical problems (RP), bodily pain (BP), general health (GH), vitality (VT), social functioning (SF), role limitations due to emotional problems (RE) and mental health (MH). In accordance with the instrument guidelines, SF-36 responses transformed as numerical values (e.g., 1–3, 1–5, or 1–6) were transformed to a 0–100 scale so that, for all items, higher scores indicated better health status. For items in which a higher response value reflected lower problem severity or fewer limitations (e.g., “never,” “not at all,” “does not limit”), forward coding was applied (e.g., 1→0 … 5→100). Conversely, for items in which a higher response value reflected greater problem severity/greater limitations (e.g., “very much,” “severe pain,” “substantially”), reverse scoring was applied (e.g., 1→100 … 5→0). For 3-point items, a 0/50/100 mapping was used, whereas for 6-point items, linear mapping to 0–100 was applied (e.g., 100/80/60/40/20/0). As a result, all items were comparable on a 0–100 scale. No missing responses were observed; therefore, no imputation was required and all domain scores were calculated directly. Then, based on the subscale scores, the PCS and MCS measures were calculated using the norm-based scoring approach: subscale scores were standardized to the reference population, weighted using the orthogonal factor scoring coefficients, and reported as T-scores (mean = 50, standard deviation = 10), with higher scores indicating better health-related quality of life, exactly as specified in the SF-36 summary scoring manual [14,15].

### 2.3. Bioethics Committee Approval

The study was conducted in accordance with the World Medical Association Declaration of Helsinki. It was approved by the Bioethics Committee of the Medical University in Białystok (APK.002.541.2023). Participants received an information sheet detailing the study, its aims, procedures, and the potential risks and benefits of participation. All participants provided written informed consent.

### 2.4. Statistical Analysis

Statistical analysis was performed to verify the research hypothesis. Quantitative variables were described using mean and standard deviation (for approximately normally distributed data) or median and interquartile range (IQR) (for non-normal distribution). Categorical variables were presented as counts and percentages.

The normality of the distribution of quantitative data was assessed using Shapiro–Wilk test and graphical methods. Depending on whether the assumptions were met, three parametric or non-parametric tests were conducted. The correlation between two quantitative variables was assessed using Spearman’s rank correlation. The differences between two independent groups were assessed using Student’s *t*-test (if the criteria were fulfilled). Differences between the three groups were assessed using one-way analysis of variance (ANOVA); in the case of a significant result, multiple comparisons were performed using the post hoc method.

In the case of lack of data, analysis was performed on the available observations for a given variable. The level of statistical significance was assumed to be α = 0.05 (*p* < 0.05). The values of test statistics and their corresponding *p* values were given in the analysis. The analysis was performed using Statistica software (version 13.3).

## 3. Results

### 3.1. Sample Characteristics

A total of 127 patients were included in the analysis. The mean age was 70.1 ± 9.2 years; men comprised 74.8% of the study group (*n* = 95) and women 25.2% (*n* = 32). The most commonly reported level of education was secondary education (66.9%, *n* = 85), while primary education was reported by 19.7% (*n* = 25) and higher education by 13.4% (*n* = 17). The data are presented in Table 3.

According to the IPAQ-SF classification, 41% of patients were categorized as having a low level of physical activity (*n* = 52), 30.7% as moderate (*n* = 39), and 28.3% as high (*n* = 36). The median total physical activity was 1022 MET-min/week [198–3183], and the median daily sitting time was 300 min/day [180–480]. The data are presented in Table 4 and Table 5.

The mean proportion of high-quality foods in the diet was 48.8 ± 13.9% (percentage of high-quality foods relative to all analyzed food items); diet quality was rated as poor in 27.6% of participants (*n* = 35), mediocre in 55.9% (*n* = 71), and good in 16.5% (*n* = 21). Vitamin supplementation was reported by 50.4% of patients (*n* = 64), and mineral supplementation by 45.7% (*n* = 58). The data are presented in Table 6.

Lipid profile data were available for 93 patients (73.2% of the sample). LDL cholesterol was analyzed in 92 patients, as in one case its concentration could not be calculated due to triglyceride levels > 400 mg/dL. The mean total cholesterol level was 153.0 ± 50.4 mg/dL, mean HDL cholesterol 44.1 ± 14.7 mg/dL, and mean triglycerides 122.6 ± 80.2 mg/dL. The mean LDL cholesterol level was 83.2 ± 40.0 mg/dL. The data are presented in Table 7.

### 3.2. Educational Level in Relation to the Proportion of High-Quality Foods in the Diet

The relationship between educational level and the quality of diet has been assessed as the percentage share of high-quality foods in diet. Data from 127 patients were analyzed.

Among patients with elementary education, mediocre diet was the most frequently reported, 56.00% (*n* = 14), followed by poor diet, 40.00% (*n* = 10). Good diet was the least frequent, reported by 4.00% (*n* = 1).

Among participants with secondary education, a mediocre diet was the most common, reported by 56.47% (*n* = 48). A poor diet was reported by 25.88% (*n* = 22) participants, and a good diet by 17.65% (*n* = 15).

Among participants with higher education, a mediocre diet was also the most common, reported by 52.94% (*n* = 9). This group also had the highest proportion of good diet recorded at 29.41% (*n* = 5), with the smallest share of poor diet at 17.65% (*n* = 3). The results are presented in Table 8.

Spearman’s rank correlation factor analysis showed a positive correlation between the educational level and the quality of the diet. This indicates that the increase in educational level is correlated to the increase in the presence of high-quality products in diet (rho = 0.281; *p* = 0.001). The obtained result shows weak, but statistically significant correlation.

### 3.3. Age in Relation to Physical Activity Levels

The relationship between age and MET value was analyzed. Analysis was done using Spearman’s rank correlation factor analysis and showed a statistically significant, moderate, negative correlation between tested variables (rho = −0.38; *p* < 0.001). The data are presented in Table 9.

### 3.4. Activity Category in Relation to Physical Component Summary (PCS)

The differences between PCS levels were analyzed in relation to the category of the activity. One-level analysis of the variance showed significant statistical differentiation in the levels of PCS between tested activity categories (F(2, 124) = 3.13; *p* = 0.047).

The highest average level of PCS was found in participants with low physical activity level (M = 33.91), with the lowest in participants engaging in mediocre physical activity (M = 30.74). Post hoc analysis showed significant differences between participants with low physical activity levels and participants with mediocre to high physical activity levels. No significant differences were found between participants engaging in mediocre and high physical activity. The data are presented in Table 10.

### 3.5. Dietary Supplement Use in Relation to Mental Component Summary (MCS)

The analysis of the mineral supplements use did not show statistically significant differences between the MCS score in patients using the supplements (M = 40.19; SD = 8.64) and those that do not (M = 41.91; SD = 7.98; t(125) = −1.16; *p* = 0.246). The data are presented in Table 11.

Differences in MCS scores in relation to dietary supplements use were analyzed. In the case of vitamin supplements, patients who did not use them showed a significantly higher level of MCS (M = 42.74; SD = 7.58) than patients who declared they used vitamin supplements (M = 39.53; SD = 8.73; t(125) = −2.21; *p* = 0.029). The data are presented in Table 12.

### 3.6. Physical Activity Level in Relation to Mental Component Summary (MCS)

A statistically significant, weak, negative correlation was found between physical activity and MCS score (rho = −0.22; *p* = 0.014). The data are presented in Table 13. 

### 3.7. Time Spent in the Sitting Position in Relation to Mental Component Study (MCS)

A statistically significant, weak, positive correlation has been found between the time spent sitting down and MCS score (rho = 0.26; *p* = 0.004).The data are presented in Table 14.

## 4. Discussion

The study analyzed the association between sociodemographic characteristics (such as age, sex, education, diet quality, physical activity and health-related quality of life) and the diagnosis of peripheral arterial disease. The quality of life was estimated by using the SF-36 questionnaire, interpreting two summary scales—physical component summary (PCS) and mental component summary (MCS). Physical activity level was assessed using the IPAQ questionnaire, calculating the value of the MET-min/week. To estimate the quality of diet, the FFQ-6 questionnaire was used. The most significant results are connected to the relationship between the quality of diet and educational level, the relationship between physical activity and age, and the differences in quality-of-life components between physical activity categories.

The hypothesis stating that the increased consumption of high-quality products in diet is associated with the increase in the educational level was confirmed (positive correlation, *p* = 0.001). This is consistent with the existing literature, in which education is considered as one of the key predictors of diet quality and dietary inequalities [16]. The higher level of education promotes higher knowledge in the field of health. It increases the understanding of the guidelines, the ability to select information and the access to higher-quality products [17]. It is important in clinical practice, as lifestyle modifications remain one of the fundamental ways to reduce the cardiovascular risks and improve the functioning of a patient with PAD. Adherence to recommendations is associated with patients’ health literacy as well as with the resources and circumstances that enable their implementation [2,8].

The hypothesis stating that the decrease in physical activity corresponds to older age in patients was confirmed, in line with epidemiological observations in the older age groups (negative correlation, *p* < 0.001). In the case of PAD, the decrease in physical activity as the patients age may be connected to the increase in pain during physical activity and the limited ability to walk. This finding is clinically relevant, as both European and American guidelines emphasize the role of personalized exercise programs (including supervised walking training) in the treatment of PAD. Additionally, the exercises aim to improve tolerance to physical strain and quality of life [2,8,18].

Testing the hypothesis that physical activity level is associated with the physical component of health-related quality of life demonstrated statistically significant differences in this component across physical activity categories (*p* = 0.047). The observed differences in the physical component of quality of life are consistent with the pathophysiology of PAD, in which pain symptoms and reduced walking distance are associated with poorer physical functioning; at the same time, walking training and structured exercise constitute important components of therapeutic management. However, the pattern of mean physical component scores—whereby the highest value was observed in the low physical activity group—warrants cautious interpretation. The observed results do not justify the conclusion that lower levels of physical activity are associated with better physical functioning. This result may be caused by the limitations inherent to a cross-sectional study and the evaluation of physical activity using the IPAQ questionnaire [19]. Physical activity can include different domains such as work, transportation, housework or leisure. Occupational physical activity does not always have health benefits and sometimes can even be detrimental, unlike leisure-time physical activity [20].

The hypothesis that using vitamin supplements has a positive effect on the MCS was not confirmed. A significantly lower MCS score was observed in the patients using vitamin supplements. It may be explained by a reverse causation effect, where individuals with poorer physical or mental health are more likely to use vitamin supplements, in attempt to improve their health [21]. In this case, the vitamin supplementation is indicative of a lower mental wellbeing, but not a cause of it, which limits the ability to interpret the results in terms of causal interpretation.

In the study population, the connection between physical activity levels and MCS supported the hypothesis taken. Higher levels of physical activity were associated with lower MCS scores. In patients with PAD, it may reflect the type of physical activity and clinical traits, rather than health-promoting characteristics of physical activity. It is possible that higher MET scores were caused by occupational or housework physical activity, performed despite pain, which could have a negative impact on mental wellbeing [20]. It may also be connected to higher physical activity due to therapeutic guidelines for PAD patients.

The hypothesis that more time spent in the sitting position is positively associated with the MCS score was proven correct. In the study group, predominantly composed of retired individuals, time spent sitting down may reflect the structure and characteristics of the usual day. Sedentary behavior may include activities such as reading, solving crosswords or watching television. These activities may positively influence mental health. According to the literature review, the connection between the behaviors connected to a sedentary lifestyle and mental health is not uniform and depends on the activities performed while sitting. Passive activities are distinguished from cognitively engaging activities, which can have different connections to symptoms of depression and wellbeing [22,23]. Consequently, the results should be interpreted as a preliminary observation, in need of further verification using multivariate analysis, considering factors such as age, educational level, progression of disease, comorbidities and the type of activities performed while sitting down.

## 5. Conclusions

The study findings indicate that lifestyle modification strategies in patients with lower-extremity peripheral artery disease (PAD) should take into account educational determinants as well as the age-related decline in physical activity. Physical activity remained significantly associated with the physical dimension of the health-related quality of life, highlighting the importance of systematic assessment and promotion of physical activity in this population. At the same time, observations regarding the mental component of quality of life suggest that increasing physical activity alone or using dietary supplementation may not necessarily be associated with better psychological wellbeing. This relationship warrants further investigation with consideration of clinical status and potential confounding factors.

Assessment of dietary habits and physical activity levels in patients with PAD provides insight into the real-world extent of deviation from recommendations and into potential barriers to their implementation. These findings may inform the planning of educational interventions focusing on diet and physical activity for patients with PAD and, consequently, may support improvements in functioning and quality of life.

A key added value of this study is the simultaneous assessment of major lifestyle factors and quality of life in a PAD population using standardized instruments. This approach facilitates comparisons across centers and may contribute useful data for future evidence syntheses (e.g., systematic reviews and meta-analyses), particularly in the context of data from the Polish population.

## Figures and Tables

**Table 1:** Conversion of FFQ-6 food-frequency categories into point weights used to construct the diet quality index.

Food Frequency Categories	Rank Scores Assigned to the Frequency Categories	Point Scores
Never or almost never	1	0
Once per quarter or less often	2	0.06
Once per month or less often	3	0.14
Several times per month	4	0.57
Once per week	5	1
Several times per week	6	2.7
Daily	7	4.5
Several times per day	8	6

(The values represent arbitrary frequency weights/points rather than the absolute number of servings per day).

**Table 2:** Criteria for classifying diet quality based on the percentage of high-quality products.

Percentage of High-Quality Products	Diet Quality Category
>60%	Good
40–60%	Mediocre
<40%	Poor

**Table 3:** Sociodemographic characteristics. Data are presented as mean ± SD or *n* (%) (*N* = 127).

Variable	*n*, (%)/SD
Age (years), mean ± SD	70.1 ± 9.2
Female	32 (25.2%)
Male	95 (74.8%)
Elementary Education	25 (19.7%)
Secondary Education	85 (66.9%)
Higher Education	17 (13.4%)

**Table 4:** Physical activity level (*N* = 127).

Category of Activity	*n* (%)
Low	52 (41%)
Moderate	39 (30.7%)
High	36 (28.3%)

**Table 5:** Physical activity and sedentary behavior (*N* = 127).

Variable	Median (IQR)
Total physical activity (MET-min/week)	1022 [198–3183]
Sitting time (min/day)	300 [180–480]

**Table 6:** Diet quality and supplementation (*N* = 127).

Variable	*n* (%)/SD
Percentage of high-quality products	48.8 ± 13.9%
Poor diet quality	35 (27.6%)
Mediocre diet quality	71 (55.9%)
Good diet quality	21 (16.5%)
Vitamin supplement use	64 (50.4%)
Mineral supplement use	58 (45.7%)

**Table 7:** Lipid profile parameters (*N* = 92 or *N* = 93).

Parameter	*n*	Mean Value ± SD
Total cholesterol (mg/dL)	93	153.0 ± 50.4
LDL (mg/dL)	92	83.2 ± 40.0
HDL (mg/dL)	93	44.1 ± 14.7
TG (mg/dL)	93	122.6 ± 80.2

**Table 8:** Correlation between the educational level and the quality of diet in tested subjects (*N* =127).

Frequency Table				
Diet Quality	Education Level *n*, (%)			Overall
	Elementary	Secondary	Higher	
Poor	10 (40%)	22 (25.88%)	3 (17.65%)	35
Mediocre	14 (56%)	48 (56.47%)	9 (52.94%)	71
Good	1 (4%)	15 (17.65%)	5 (29.41%)	21
Overall	25	85	17	127

**Table 9:** Correlation between age and MET value (*N* = 127).

Variable	*n*	Rho	*p*
Age—MET sum	127	−0.38	<0.001

**Table 10:** PCS level in relation to physical activity categories (*N* =127).

Category of Activity	*n*	M	SD
Mediocre	39	30.74	5.75
Low	52	33.91	6.88
High	36	31.69	5.77
F(2, 124) = 3.13; *p* = 0.047			

**Table 11:** MCS score in relation to the mineral supplements use (*N* = 127).

Mineral Supplements	*n*	M	SD	t	df	*p*
Yes	58	40.19	8.64	−1.16	125	0.246
No	69	41.91	7.98			

**Table 12:** MCS score in relation to the vitamin supplements use (*N* = 127).

Vitamin Supplements	*n*	M	SD	t	df	*p*
Yes	64	39.53	8.73	−2.21	125	0.029
No	63	42.74	7.58			

**Table 13:** MCS score in relation to physical activity (MET) (*N* = 127).

Variables	*n*	Rho	*p*
MET sum—MCS	127	−0.22	0.014

**Table 14:** Time spent in the sitting position in relation to MCS score (*N* = 127).

Variables	*n*	Rho	*p*
Sitting (min/day)—MCS	127	0.26	0.004

## Data Availability

Data is contained within the article.

## Abbreviations

The following abbreviations are used in this manuscript:

PAD	Peripheral arterial disease
MCS	Mental component summary
PCS	Physical component summary
IPAQ	Linear dichroism
FFQ-6	Food frequency questionnaire
SF-36	36-item short form survey
MET	Metabolic equivalent of task
LDL	Low-density lipoprotein
HDL	High-density lipoprotein
PF	Physical functioning
RP	Role limitations due to physical problems
GH	General health
VT	Vitality
SF	Social functioning
RE	Role limitations due to emotional problems
MH	Mental health
ANOVA	Analysis of variance
*n*	Sample size for a given analysis/subgroup (number of observations)
rho	Sperman’s rank correlation coefficient
*p*	*p*-value (level of statistical significance)
SD	Standard deviation
M	Arithmetic mean
t	The t statistic from Student’s t-test
df	Degrees of freedom associated with a test statistic
BP	Bodily pain
*N*	Total sample size (overall number of participants)
IQR	Interquartile range
